# A BAC-based physical map of *Brachypodium distachyon *and its comparative analysis with rice and wheat

**DOI:** 10.1186/1471-2164-10-496

**Published:** 2009-10-27

**Authors:** Yong Q Gu, Yaqin Ma, Naxin Huo, John P Vogel, Frank M You, Gerard R Lazo, William M Nelson, Carol Soderlund, Jan Dvorak, Olin D Anderson, Ming-Cheng Luo

**Affiliations:** 1 Genomics and Gene Discovery Research Unit, USDA-ARS, Western Regional Research Center, 800 Buchanan Street, Albany, CA 94710, USA; 2 Department of Plant Sciences, University of California, Davis, CA 95616, USA; 3 BIO5 Institute, University of Arizona, Tucson, AZ 85721, USA

## Abstract

**Background:**

*Brachypodium distachyon *(*Brachypodium*) has been recognized as a new model species for comparative and functional genomics of cereal and bioenergy crops because it possesses many biological attributes desirable in a model, such as a small genome size, short stature, self-pollinating habit, and short generation cycle. To maximize the utility of *Brachypodiu*m as a model for basic and applied research it is necessary to develop genomic resources for it. A BAC-based physical map is one of them. A physical map will facilitate analysis of genome structure, comparative genomics, and assembly of the entire genome sequence.

**Results:**

A total of 67,151 *Brachypodium *BAC clones were fingerprinted with the SNaPshot HICF fingerprinting method and a genome-wide physical map of the *Brachypodium *genome was constructed. The map consisted of 671 contigs and 2,161 clones remained as singletons. The contigs and singletons spanned 414 Mb. A total of 13,970 gene-related sequences were detected in the BAC end sequences (BES). These gene tags aligned 345 contigs with 336 Mb of rice genome sequence, showing that *Brachypodium *and rice genomes are generally highly colinear. Divergent regions were mainly in the rice centromeric regions. A dot-plot of *Brachypodium *contigs against the rice genome sequences revealed remnants of the whole-genome duplication caused by paleotetraploidy, which were previously found in rice and sorghum. *Brachypodium *contigs were anchored to the wheat deletion bin maps with the BES gene-tags, opening the door to *Brachypodium*-Triticeae comparative genomics.

**Conclusion:**

The construction of the *Brachypodium *physical map, and its comparison with the rice genome sequence demonstrated the utility of the SNaPshot-HICF method in the construction of BAC-based physical maps. The map represents an important genomic resource for the completion of *Brachypodium *genome sequence and grass comparative genomics. A draft of the physical map and its comparisons with rice and wheat are available at .

## Background

Model systems play an important role in studies of genome structure and evolution, and are invaluable in gene isolation and functional characterization. The application of model systems toward the study of both basic and applied problems in plant biology has become routine. The model dicot *Arabidopsis thaliana *has been used in studies ranging from nutrient uptake and metabolism to plant-pathogen interactions. Unfortunately, due to its distant relationship to monocots, Arabidopsis is not an ideal model for grasses. Rice is being currently used as a grass model [1], but its primary adaptation to semi-aquatic, subtropical environments limits its usefulness. The large sizes of rice plants and long generation time make experiments requiring large numbers of plants grown under controlled conditions costly. It is also challenging to grow rice under the conditions prevailing in greenhouses in northern climates.

*Brachypodium distachyon *has numerous attributes expected to find in a genetic model and interest in using it as a model system for wheat and other temperate grasses is growing rapidly [2-8]. Diploid *B. distachyon *is closely related to the Triticeae [9,10] but in contrast to the Triticeae, it possesses a very small genome (x = 5) of approximately 355 Mb [9,11]. The recent release of 8× *B. distachyon *genome sequence showed that the genome is 271 Mb in size (assembled sequences, ). It is a small temperate grass with simple growth requirements, short generation time, and self-pollinating habit [2,6,7,9]. Highly efficient transformation of *B. distachyon via Agrobacterium tumefaciens *has been developed, which will facilitate its functional genomics and biotechnological applications [12-14]. These characteristics make *B. distachyon *superbly suitable for both functional and comparative genomic research.

Several genomic regions of *B. distachyon *and *B. sylvaticum*, a close relative of *B. distachyon *with a larger genome, have been compared with wheat and rice. In general, good colinearity was observed reflecting general conservation of synteny across the grass family [15-19]. To foster the development of *B. distachyon *as a grass model and coordinate the development of its genomics resources, the International Brachypodium Initiative was formed . The Initiative placed a high priority on the development of a global physical map of diploid *B. distachyon *composed of large genomic fragments cloned in a bacterial artificial chromosome vector (BAC) . A high resolution BAC-based physical map has many genomics applications including analyzing genome structure, conducting genome-wide comparisons, and facilitating the assembly of *B. distachyon *genome sequence.

The development of a *Brachypodium *BAC-based physical map is reported here. Also reported is a global comparison of the map with rice genome sequence [1] and wheat deletion bin maps [20] with the goal to obtain a clearer picture of *B. distachyon *genome structure and evolutionary history and their relationships to those of rice and wheat.

## Results and Discussion

### BAC source, fingerprinting, and contig assembly

**Figure 1:**
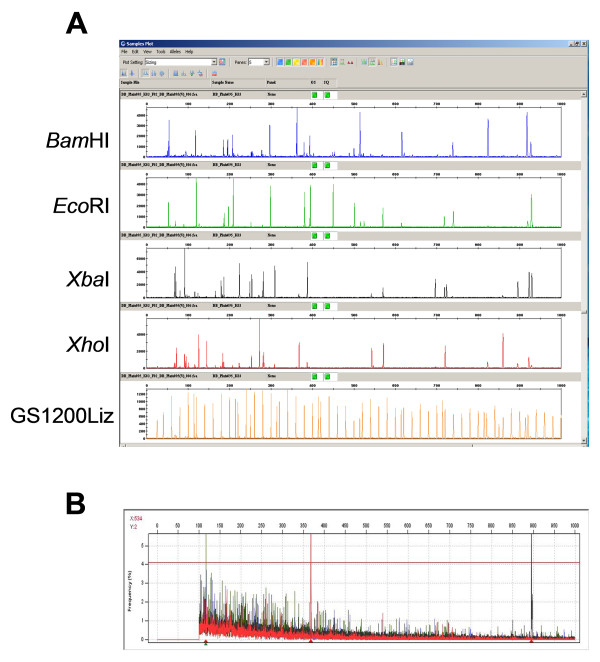
**Fragment sizing with ABI 3730xl and frequency distribution of fragment sizes using GS1200Liz size standard**. Figure 1A shows an example of fingerprinting profile of a digested BAC clone using GS1200Liz as a size standard. The fingerprinting of each BAC involved digestion with five restricted enzymes and labeling with four fluorescent dyes as described previously [22]. The size for each fragment was calculated based on co-migration of size standard in the capillary. Figure 1B shows the frequency of fragments with different sizes in 14,231 fingerprinted *Brachypodium *BAC clones. Large peaks represent vector fragments that appear in high frequencies. The red line defines the threshold for high frequency fragments derived from BAC inserts. Fragments with a frequency above the threshold were removed prior to contig assembly due to their likely origin from repetitive sequences.

A total of 67,151 clones of *Hin*dIII and *Bam*HI BAC libraries developed from the diploid *B. distachyon *accession Bd21 [21] were fingerprinted using the SNaPshot HICF BAC fingerprinting method [22,23]. To generate more information about each clone, a GS1200Liz size standard, which allows sizing of restriction fragments up to 1,000 bp (Figure 1A), was used. The use of GS1200Liz necessitated using the 50-cm capillary array for the ABI 3730XL, instead of the standard 36-cm capillary array that is used for electrophoresis of fragments ranging from 50 bp to 500 bp [22,24,25]. Large-size fragments are less frequent than small-size fragments in the SNaPshot HICF profiles (Figure 1B), and are more valuable in contig assembly because they are less likely to be shared by chance [22]. Since more large fragments could be called using the GS1200Liz as size standard, fragments with size less than 100 bp were not used for contig assembly in this study.

Cross-contamination and low quality fingerprinting data interfere with accurate contig assembly [24]. Contaminated clones, empty clones, small insert clones, and clones with fingerprints below specified quality threshold were eliminated with the GenoProfiler program [26]. Of the 67,151 fingerprinted clones, 52,343 clones (78%) were suitable for contig assembly. An average fingerprint had 79.4 restriction fragments in this population of fingerprints. Since the average insert size was 100 kb [21], there was on the average a restriction fragment every 1.26 kb.

The 52,343 fingerprints representing 14× *B. distachyon *genome equivalents were used for an initial automated contig assembly using the FPC software [27]. The initial assembly was performed at a relatively high stringency (1 × 10^-45^) to minimize faulty contig assembly of clones from unrelated regions of the genome. The "DQer" function was used to dissemble contigs containing more than 10% questionable (Q) clones. The "End to End" FPC function was then repeatedly employed to merge contigs with successively less stringent Sulston score cutoff values [24,25,28]. In the end, the FPC assembly resulted in 648 contigs containing a total of 50,182 BAC clones. In this "Phase I" physical map, 177 contigs had more than 100 clones each, 73 contigs had 50 - 99 clones each, 72 contigs had 10 - 49 clones, and the rest had 9 clones or less. A total of 2,161 singletons remained. The cumulative, contiguous, non-redundant fragment count across all contigs was equivalent to approximately 410 Mb, which was 15.5% more than the estimated size of *B. distachyon *genome (355 Mb) [9,11]; if the genome size of 271 Mb based on the recent release of 8× genome sequence assembly  is used, the fragment count would be equivalent to 51.3% more of the estimated genome size. This indicated that many contigs actually overlapped other contigs, but the overlaps were below contig joining threshold. Such overestimation has been reported in physical maps of other plant genomes [25,29].

### Editing of contigs by alignments with the rice genome sequence

Integration of molecular markers into contigs is crucial for their anchoring on genetic maps and ultimate alignment of a physical map and genome sequence. This task can be accomplished by screening BAC libraries with pools of labeled probes derived from EST clones or mapped genetic markers or screening of multidimensional pools of BAC clones by PCR or highly parallel Illumina GoldenGate assays [30-33]. BAC end sequences (BESs), in addition to other genomic applications [34-37], can facilitate initial genome characterization [3,28,34,35] and anchoring of contigs onto the genetic map. BESs are particularly useful for contig anchoring in small, gene-dense genomes. Their utility is diminished in large and complex genomes due to a low gene density. For example, in wheat, over 80% of the genome consists of repetitive DNA (reviewed in [38]). Akhunov *et al*. [39] reported that coding sequences accounted for only 5.8, 4.5, and 4.8% of BES in *T. uratu*, *Ae. speltoides*, and *Ae. tauschii *BAC libraries, respectively. A total of 38 Mb of random *B. distachyon *genomic sequence was generated by sequencing 64,694 BAC ends from the two BAC libraries, representing ~14.0% of the genome sequence on the basis of a genome size of 271 Mb . This was equivalent to one sequence tag every 4.2 kb (considering 271 Mb of the genome size). A total of 25.3% of repeat-masked *B. distachyon *BESs had matches to the rice genome sequence (*E *< 10^-25^). Among them, 13,970 also matched wheat ESTs [3]. Therefore, the integration of *B. distachyon *BES into the contigs immediately anchored a large number of contigs onto the rice genome sequence and wheat deletion maps (see discussion below).

**Figure 2:**
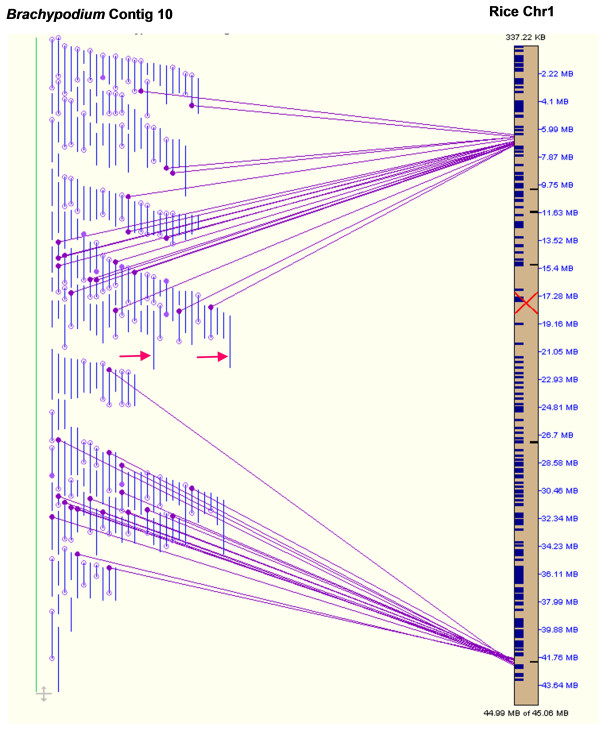
**The SyMAP close-up view shows the false joining of contigs caused by clone contamination**. Contig 10 from the Phase I assembly matched two rice regions that were separated by over 35 Mb on rice chromosome 1 (Chr1). Solid vertical lines represent BAC clones. Dots at the ends of solid vertical lines represent BESs generated for the corresponding BAC clones. Empty dot represents BES with no significant match to the rice genome. The dots connected by lines indicate that the BESs have matches in the corresponding orthologous positions in the rice genome. Filled dots with no connecting lines indicate BESs with matches to rice sequences located in different regions of the rice genome. Two cross-contaminated clones that caused false joining of the two clusters are indicated by arrows (not part of the SyMAP display).

BES of fingerprinted clones facilitated manual editing and contig assembly validation. This was based on the assumption that closely related grass genomes share extensive colinearity. The colinearity of contigs with the rice genome can be used to assess quality of SNaPshot-based BAC fingerprinting technology and contig assembly. *Brachypodium *contigs with BESs allowed for direct alignment of contigs with rice pseudomolecules; BLAT [40] was used for finding sequence similarities, which were then used by SyMAP (Synteny Mapping and Analysis Program [41]) for computing the synteny blocks and visualizing the results (Figure 2 and results below). These alignments were used to guide contig editing and disjoining, as it was inevitable that miss-assembled BAC contigs occurred due to a number of factors including chimeric clones and cross-contamination. In addition, contig merging was performed with successively increasing cutoffs (as high as 1 × 10^-14^), so it was likely that some merging could result in false joining of two unrelated regions. We used alignments with the rice genome as reference to provide supporting evidence during disjoining problem contigs. During contig editing, when two merged contigs aligned to two different regions in the rice genome, the merge was rejected and the merged contigs were disjoined. The same strategy can be applied to miss-assembled contigs. When a contig is aligned to different rice genomic regions, the contig should be further evaluated to identify potential assembly problems. For example, in the initial assembly, Contig10 was aligned to two genomic blocks on rice chromosome 1, separated by over 35 Mb (Figure 2). It was found that the contig contained two clusters linked by two BAC clones, DB064D23 and DB064F23. These two clones reside near each other in a 96-well plate, indicating that cross-contamination may have occurred during fingerprinting process (inoculation or transfer) and probably resulted in two shared fingerprint profiles just below the predefined contamination threshold. Contig 10 was disjoined into two after removing the two clones during the contig editing process.

**Figure 3:**
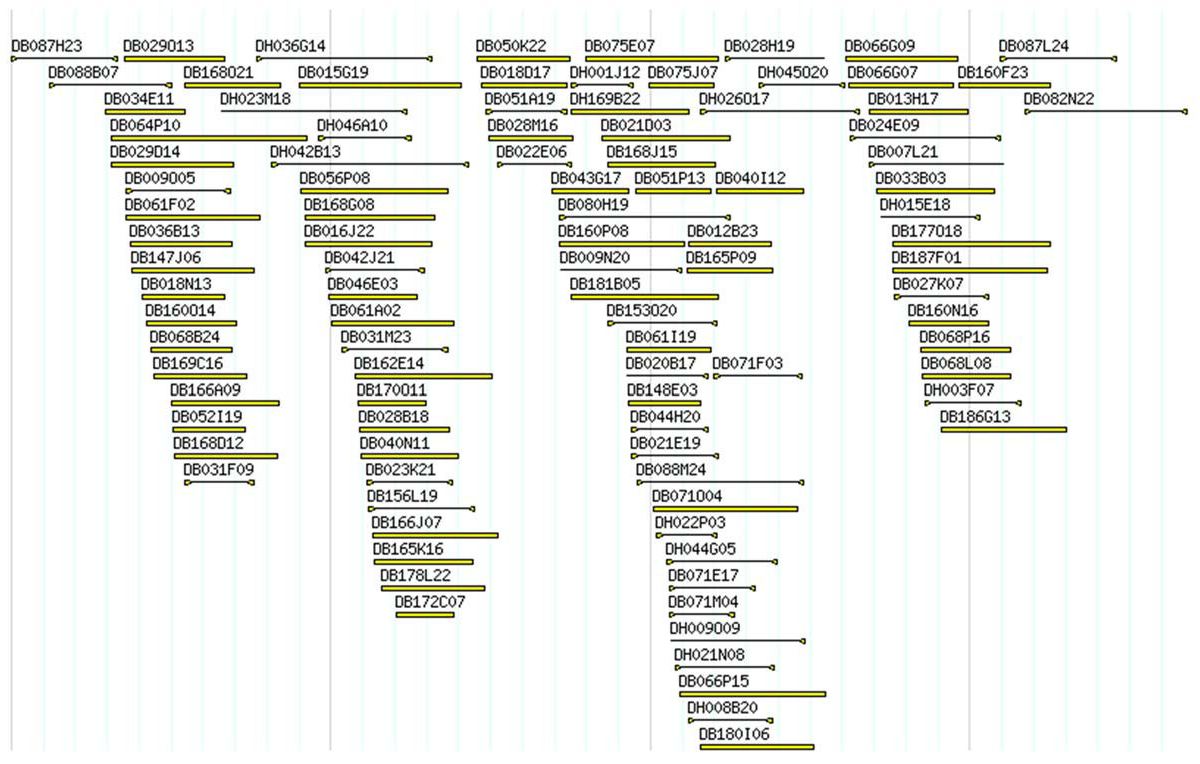
**FPC contig view of a *Brachypodium *contig**. *Brachypodium *Contig133 is used as an example. Clones with solid black lines below the clone name represent those with BES. A triangle at both ends of the line indicated that both ends of the BAC clone were sequenced, while a triangle at one end indicates that BES is only present at that end. The clones prefixed with "DH" and "DB" were from *B*. *distachyon Hin*dIII and *Bam*HI BAC libraries, respectively.

The integration of BES into contigs and manual editing of contigs using rice genome as a reference improved contig assembly by disjoining 23 contigs. The final assembly contained 671 contigs, which included BESs. This assembly is called "Phase II physical map" of the *B. distachyon *genome. Figure 3 shows an example of a contig in the Phase II physical map. The view of the complete set of *B. distachyon *contigs is available at .

### Comparison of *B. distachyon *contigs with the rice genome

The alignment of contigs of the Phase II *B. distachyon *physical map to the rice genome sequence estimated the genome coverage. A total of 345 contigs (51.4%) could be aligned to the rice genome sequence. They covered 336 Mb (88%) of the rice genome sequence (using 382 Mb as 1*C *rice genome size, [1]) and represented 88% of the total *B. distachyon *FPC map as measured by CB units. When only contigs with more than 10 clones were used, 331 out of 364 (90.9%) could be aligned to the rice genome. Although 326 contigs could not be anchored, these contigs were generally small, and the total number of clones in them equaled to only 2,489 (5.0%) out of the total 50,182 clones, indicating that only a small portion of the clones could not been anchored onto the rice genome. The data suggested a general conservation of synteny between rice and *B. distachyon *genomes, which confirms previous conclusions made on the basis of sequencing a few *B. sylvaticum *BAC clones and their sequence comparisons with the orthologous regions in rice and wheat [15].

**Figure 4:**
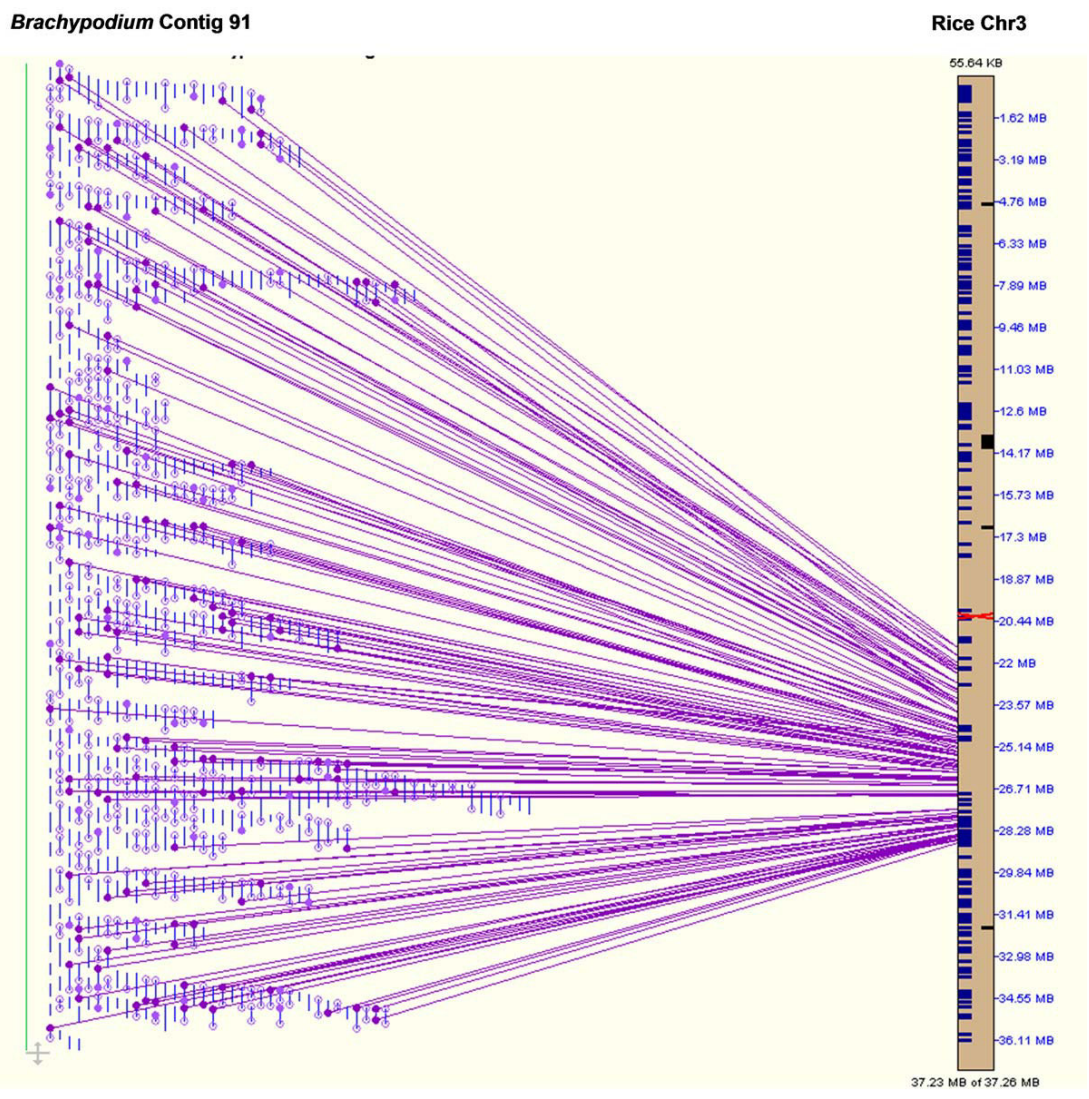
**Colinearity of *Brachypodium *Contig 91 with a 7-Mb genomic region on rice chromosome 3 (Chr3)**. Contig 91 contains 678 BAC clones with a total of 549 BES. This contig aligned to a 7-Mb genomic region on rice Chr3 based on BLAT comparison. BESs that match rice sequence in the 7-Mb orthologous region were connected to the corresponding position with a solid line.

Ideally, *B. distachyon *contigs should be ordered using a high-resolution genetic map. Such a map was not available to us. However, the SyMAP alignment of the FPC contigs onto the sequenced rice chromosomes ordered many of the contigs into synteny blocks with putative chromosome assignments and showed extensive colinearity of contigs with the rice genome sequences. For instance, Contig 91 appeared to be highly colinear with a 7-Mb region in rice chromosome 3 although several small local inversions could be identified (Figure 4). Out of 160 BESs in this contig homologous to the rice genome sequence, 117 (73%) were homologous to sequences in this region on rice chromosome 3, while 53 were homologous with sequences in different regions of the rice genome, presumably representing non-colinear genes. Previous alignment of ten sequenced *B. distachyon *BAC clones with the orthologous rice regions also revealed a general synteny conservation between rice and *B. distachyon *[19]. It was found that 15% *B. distachyon *and 19% rice genes were not present in the corresponding orthologous regions [19]. The non-colinear genes reflect the divergence of the two genomes after the split from a common ancestor during phylogeny of the grass family [42].

**Figure 5:**
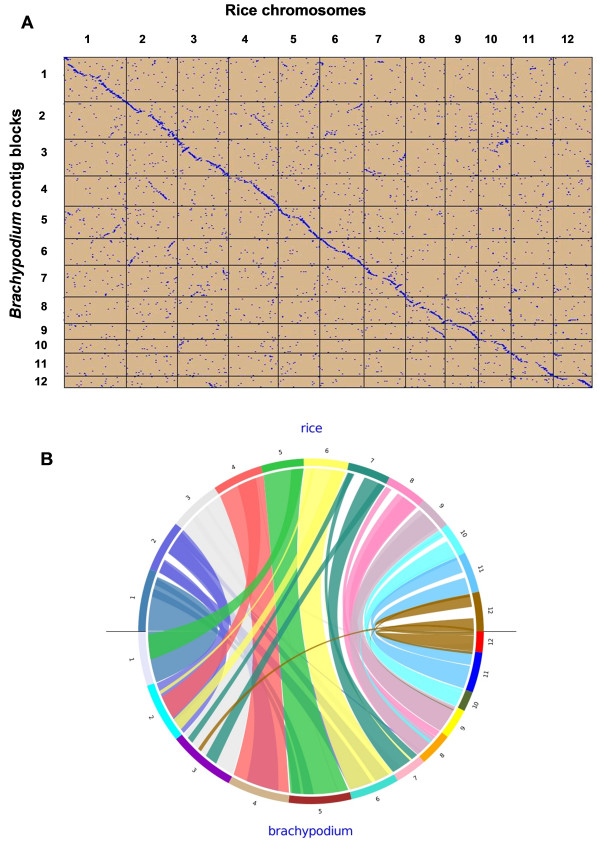
**Comparative analyses of *Brachypodium *contigs against rice genome**. Figure 5A. The SyMAP dotplot analysis of *Brachypodium *contig blocks against rice genome. The *Brachypodium *contigs were first aligned to 12 rice chromosomes based on BLAT analysis to generate 12 *Brachypodium *synteny contig blocks. These 12 *Brachypodium *synteny blocks were then compared with the pseudomolecules of rice chromosomes using dotplot analysis. Synteny blocks were detected, and background noise was filtered with SyMap [41]. Figure 5B. Ancient duplication within the rice and *Brachypodium *genomes. Evidence of ancient duplication can been seen when the rice chromosome is colinear to two *Brachypodium *contig blocks as highlighted by colored lines in the two genomes.

A SyMap dot-plot (Figure 5A) shows correspondence of most *B. distachyon *contigs to specific regions of the rice genomic sequence. This is consistent with the estimation that the *B. distachyo*n contigs cover 336 Mb or ~88% of the rice genome. A question remains whether or not these alignments provide evidence of general colinearity between rice and *B. distachyon *chromosomes. Although the *B. distachyon *contigs are not ordered, the following line of reasoning suggests that the *B. distachyon *and rice chromosomes are highly collinear. Differences in gene order between *B. distachyon *and rice due to inversions or translocations would be detected in *B. distachyon *BAC contigs as breaks in co-linearity not associated with week joins . Fourteen such genuine breaks in colinearity are expected between the two genomes reflecting the difference in chromosome number; *x *= 12 in rice to *x *= 5 in *B. distachyon*. Of the 364 contigs having more than 10 clones (see above), 33 may have such colinearity breaks. If 14 are due to chromosome number differences, this leaves only 19 contigs, or 5% of the total 364 contigs to be potentially due to actual or artifactual breaks in colinearity. Since there is no compelling reason why breaks in gene colinearity should coincide with gaps between BAC contigs, we therefore conclude that *B. distachyon *and rice chromosomes are highly colinear and most of the neighboring contigs on the dot-plot alignment in Figure 5A are good candidates for joining.

Most plant genomes are paleopolyploid with ancient whole-genome duplications [43-48]. The radiation of grasses was preceded by paleotetraploidy resulting in a whole-genome duplication [46], which was followed by diploidization by deletions. Therefore, in regions that are still duplicated in the *B. distachyon *genome, a rice region will align strongly with one *B. distachyon *contig block and weakly with another. This is evident in Figure 5B, where two *B. distachyon *syntenic contig blocks can be identified for several rice chromosomes. These data show that the *B. distachyon *genome has a similar set of duplications derived from the ancient paleotetraploid as does rice.

### Alignment of wheat EST deletion bin map to *B. distachyon *contigs

The 1C nucleus of hexaploid *T. aestivum *contains 16,000 Mb DNA [49], which makes map-based cloning of wheat genes very difficult. Because *B. distachyon *diverged from the wheat phylogenetic lineage only about 30 million years ago (MYA) [15], *B. distachyon *physical map and ultimately genome sequence can facilitate wheat map-based cloning and other genomic applications.

A total of 7,104 expressed sequence tag (EST) unigenes were previously mapped into 156 deletion bins, providing a genome-wide framework for wheat mapping and identifying agronomically important genes [20]. However, a disadvantage of the deletion bin map is that loci are not ordered within the bins. Given the general colinearity among the grass genomes, this problems can be partially overcome by *in silico *ordering of wheat ESTs using rice genomic sequence [50]. *Brachypodium *is expected to show better synteny with wheat than rice because it diverged from wheat more recently than rice [3], *Brachypodium *is therefore expected to be more useful in comparative mapping applications than rice.

To assess the utility of *B. distachyon *physical map for wheat genomics, we compared the *Brachypodium *contigs with the wheat deletion bin map [20]. Of 7,104 deletion bin mapped wheat ESTs, 985 matched *Brachypodium *BESs at an e-value cutoff of 1× 10^-10^. These matches were derived from BESs associated with 216 contigs (32% of total *Brachypodium *contigs). Such analysis allowed us to align *Brachypodium *contigs onto individual chromosomes based on wheat deletion bin map data .

**Figure 6:**
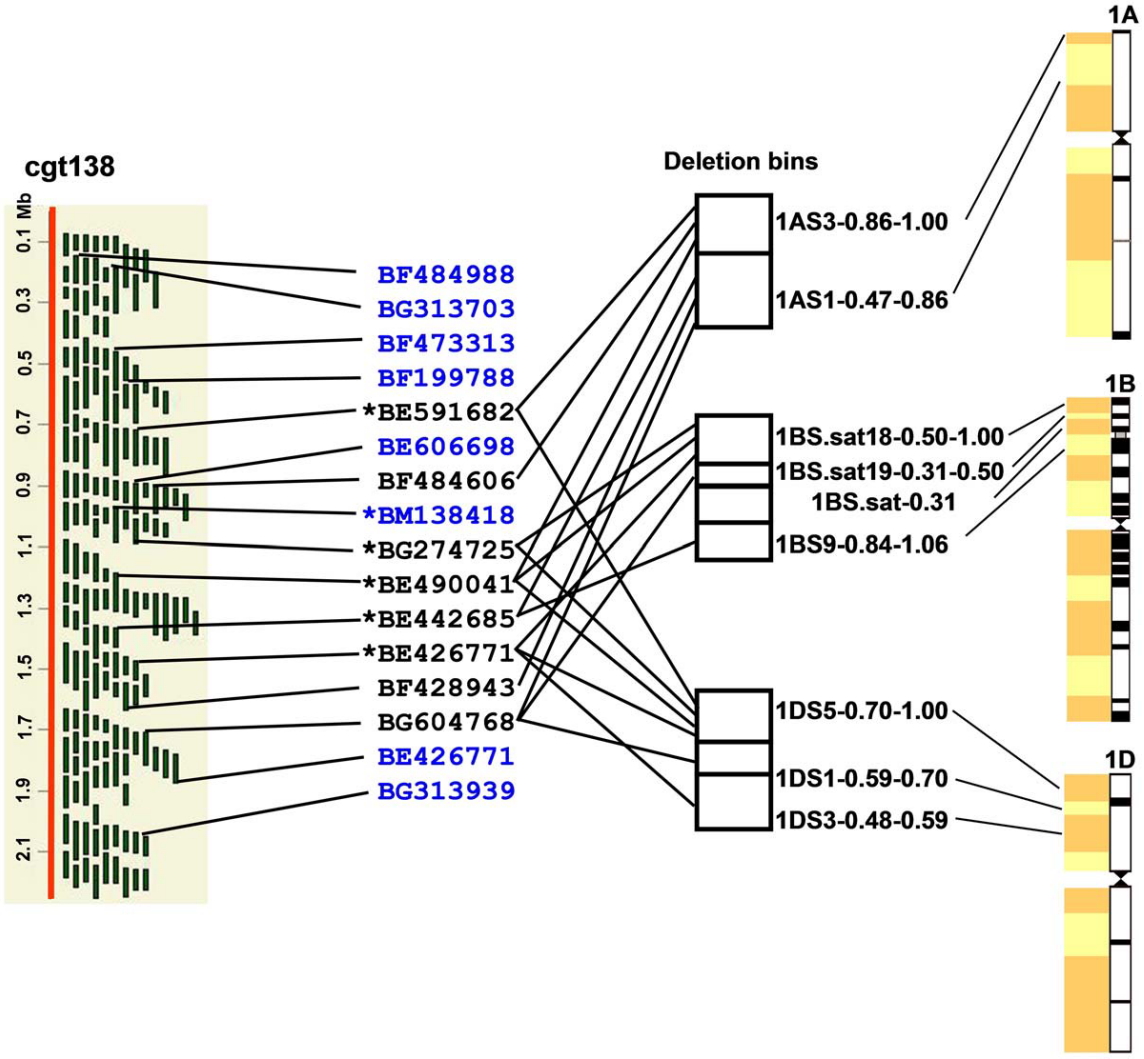
**Anchoring *Brachypodium *contigs to wheat deletion bins**. *Brachypodium *Contig138 is orthologous with a genomic region on rice chromosome 5 based on BLAST search. A total of 16 mapped wheat ESTs were matched by the BES in the contig. The ESTs were connected to the contig by a line from only one representative BAC end. Eight ESTs in black were mapped to deletion bins located at the distal regions of wheat group 1 chromosomes. The bin location for each EST was indicated by a black line. A schematic drawing of wheat group 1 chromosomes and associated deletion bins as indicated by colored boxes are provided to specify the fraction lengths of wheat chromosome bins. ESTs in blue represent those that were mapped to different regions in the wheat genome. Colinear ESTs between rice and *Brachypodium *are indicated by a star. BAC clones are indicated by a solid vertical bar in Contig138. The approximate distance along the contig was calculated based on CB units and is indicated in Mb.

Comparison of *Brachypodium *contigs with the wheat deletion bin map and the rice genome sequence provided a genome-wide view of genome evolution among these species. Figure 6 shows an example of such a comparison. Contig138 contained 1,380 BAC clones with 1,402 BESs and spanned 2.2 Mb (estimated from a relationship between CB units and Mb). A total of 16 wheat ESTs in the deletion bins matched BESs in this contig. Eight of them were mapped in the distal bins 1AS3-0.86-1.00, 1AS1-0.47-0.86, 1BS.sat18-0.50-1.00, 1BS.sat19-0.31.0.50, 1BS.sat19-0.31-0.50, 1BS.sat-0.31, 1BS9-0.84-1.06, 1DS5-0.70-1.00, 1DS1-0.59-0.70, and 1DS3-0.48-0.59 on wheat chromosomes 1A, 1B, and 1D [20] (Figure 6). They were distributed across two to three bins on each chromosome, indicating that the contig spans multiple bins on wheat group 1 chromosomes (Figure 6). In most cases, the order of BESs in a contig homologous to wheat ESTs correlated with the order of bins along a chromosome arm into which these ESTs were mapped. These data suggested that Contig138 represented an orthologous region of these deletion bins. Alignment with rice genome indicated that Contig138 was colinear with a region on rice chromosome 5. Among eight colinear wheat ESTs between *Brachypodium *and wheat, three ESTs (BF484606, BF428943, and BG604768) were not colinear with the rice orthologous region. BF428943 and BG604768 were homologous to genes on rice chromosome 2, suggesting gene duplication/deletion either in rice or in the Pooideae lineage after Pooideae diverged from Ehrhartoideae but before divergence of *Brachypodium*. No match to BE484606 was found in the rice genome based on BLAST searches.

ESTs BF473313 and BE446475 homologous to BES sequences of Contig138 also have no match in the rice genome, but were mapped on long arms of wheat chromosomes of homeologous groups 1 and 7, respectively. These genes are only present in *Brachypodium *and wheat but are not located in colinear positions in the two genomes. Another EST locus, BM138418, is colinear only between *Brachypodium *and rice since this EST was mapped to the chromosome 3B in wheat based on the wheat deletion bin map result. Two ESTs, BF484988 and BG313703, at the top of Contig138 were mapped to the short arm on wheat chromosomes of homeologous group 5. In rice, they matched sequences on chromosome 1. Other examples of noncolinear genes among the three genomes were observed. Occasional perturbations of synteny between *Brachypodium*, rice and wheat must therefore be expected and taken into account in comparative genomic applications. Since synteny of wheat chromosomes has been eroded faster in the distal regions than in proximal regions [51], perturbed synteny should be expected particularly in genomic comparisons with *Brachypodium *involving distal regions of wheat chromosomes. Synteny perturbations observed in contig 138 exemplify this reality since the contig is orthologous to a distal region of the short arm of chromosome 1.

To further expand the utility of the *Brachypodium *physical map, we integrated wheat ESTs that have not been placed into wheat deletion bins into *Brachypodium *contigs. There are over one million wheat ESTs in Genbank, and they have been assembled into 42,848 unigene sets . Using BLAT, 5,421 different unigenes were integrated to the *Brachypodium *contigs. They were homologous to a total of 5,910 BES in the physical map. These wheat EST markers will further facilitate comparative mapping in wheat. The comparison of *Brachypodium *contigs with wheat deletion maps and integration of wheat unigenes into *Brachypodium *contigs can be accessed at .

## Conclusion

A whole-genome, BAC-based physical map for the new grass model species, *Brachypodium*, was constructed. The map facilitated a variety of genomic applications. Comparison of contigs with rice revealed high colinearity between rice and *Brachypodium *chromosomes. Additionally, many *Brachypodium *BAC contigs could be anchored on the wheat deletion bin maps. Both outcomes support the anticipated utility of *Brachypodium *for wheat comparative genomics and practical applications in map-based cloning of wheat genes. Another application of the physical map is in the *Brachypodium *genome sequencing project. The phase II physical map can be aligned with shotgun sequence contigs *via *BES and provide scaffolds for ordering shotgun sequence contigs and estimating gap sizes. The BAC-based physical map reported here was provided to the *Brachypodium distachyon *sequence assembly team and has helped produce a high quality final sequence assembly . The physical map can also identify BACs spanning sequence gaps and has served as sequencing template to fill them. *Brachypodium *is expected to serve as a surrogate to assist gene discovery and functional characterization in the large and complex crop genomes, such as wheat and bioenergy crops. All the data and resources developed in this study are available at the  website for community use.

## Methods

### Bacterial artificial chromosomal (BAC) libraries

The *B. distachyon *BAC libraries used in this study were previously constructed using partial digests with *Hin*dIII and *Bam*HI restriction enzymes from an inbred diploid line of *B. distachyon*, Bd21, the same line selected for complete sequencing by the US Department of Energy Joint Genome Institute through its "Community Sequencing Program" [21]. These BAC libraries represent ~29.2-fold haploid genome equivalents and were employed for BAC fingerprinting and contig assembly.

### BAC fingerprinting and fragment sizing

The BAC clones were fingerprinted as described by [22] with minor modifications. From each 384-well plate, four 96-well blocks containing 1.2 ml of 2× YT medium plus 12.5 μg/ml chloramphenicol were inoculated with a 96-well replicator. Two pins were removed from the replicator to allow the insertion of control clones into the 96-well plate. Two control BAC clones were inserted manually in wells E07 and H12 in each 96-well block. The plates were covered with Airpore gas permeable plate sealant (Qiagen) and shaken on an orbital shaker agitated at 400 rpm at 37°C for 20 hours. BAC DNA was isolated with the Qiagen R.E.A.L 96-Prep kit (Qiagen, Valencia, California). The following minor modifications of the fingerprinting method were made to accommodate the use of ABI3730XL (Applied Biosystems, Foster City, California) instead of ABI3100 for capillary electrophoresis. The more sensitive laser of the ABI3730XL instrument improved fingerprinting resolution and made it possible to reduce the amount of BAC DNA sample for electrophoresis, thus lowering fingerprinting costs. To reduce sample size, 0.5-1.2 μg instead of 1.0-2.0 μg of BAC DNA was simultaneously digested with 2.0 instead of 5.0 units each *Bam*HI, *Eco*RI, *Xba*I, *Xho*I and *Hae*III (New England Biolabs, Beverly, Massachusetts) at 37°C for 3 hrs. The DNA was labeled with 0.4 μl instead of 1.0 μl of the SNaPshot kit (Applied Biosystems, Foster City, California) at 65°C for 1 hr and precipitated with ethanol. The labeled DNA was dissolved in 9.9 μl of Hi-Di formamide, and 0.3 μl of GeneScan 1200 LIZ (Applied Biosystems, Foster City, California) was added to each sample as an internal size standard. Restriction fragments were sized with ABI3730XL using 50 cm capillaries and POP7 (Applied Biosystems, Foster City, California). The fragment size calling was accomplished with the GeneMaper software (Applied Biosystems, Foster City, California) with the help of FP Pipeliner .

### Fingerprints editing and contig assembly

The fingerprint profiles for each BAC clone were collected by GeneMapper v3.7 (Applied Biosystems). The GeneMaper output data was edited with the GenoProfiler program [26]. The two control BAC clones inserted in each 96-well plate were used to check for the correct orientation of the plate. Fragments in the size range of 100 - 1,000 bp were measured. For the data quality control, vector bands and clones failing fingerprinting or lacking inserts were removed using GenoProfiler program. In addition, samples with less than 40 or more than 150 fragments were also eliminated. Fingerprints of cross-contaminated samples were detected using a module in the GenoProfiler [26] and removed from the data set. The cross-contamination was defined as clones residing in neighboring wells either in 384-well format or 96-well format (quadrants) share 30% or more of the mean number of fragments, the formula is shared bands*2/(bands1 + bands2).

A total of 52,343 BAC clones that passed quality check were used to assemble contigs using the FPC v8.5.3 [27]. The initial assembly used a Sulston score of 1× 10^-45 ^and a tolerance 0.4 bp. The contig assembly was processed through the DQ function of FPC to break up contigs having more than 10% Q-clones in a contig. The assembly was further refined using "Single-to-End" and "End-to-End" merging by stepwise decreasing of assembly stringency of Sulston score cutoff values (down to 1× 10^-14^).

### Alignment of FPC contig with the rice genome and wheat deletion bin map

There were 64,697 BAC end sequences generated from the fingerprinted *B. distachyon *BAC clones [3] which were compared using BLAT [40] against the rice pseudomolecules build 4.0 . The results were used as input into Synteny Mapping and Analysis Program (SyMap) [41], which computed the synteny blocks. The results can be viewed from the SyMAP Java based dot-plot, synteny block to chromosome, and close-up views.

To anchor *B. distachyon *physical contigs to the wheat deletion bin map, BAC-end sequence data from the *B. distachyon *BACs were compared against sequences of the mapped wheat EST probe sets using BLAT (parameters minScore = 50, minIdentity = 80%) and links to the wheat deletion bin map constructed [20,52]. The BLAT alignments and FPC anchoring were carried out using FPC modules BSS, which is designed for this purpose [53]. The CMAP  tool allowed for comparative analysis between the *B. distachyon *physical contigs and wheat deletion bin map.

## Authors' contributions

YQG, YM, NH, and JPV isolated BAC DNA. YM generated the fingerprinting data. MCL, FMY, and NH analyzed fingerprints, assembled the physical map, and verified assembly. FMY, MCL, GRL, WMN, and CS developed database and interfaces to display FPC results on the web and performed comparative studies. YQG and MCL conceived the project, supervised its execution, and draft the manuscript. JPV, JD, and ODA contributed to the planning of the project and edited the manuscript. All authors read and approved the final version of the manuscript.
